# Influence of air intake from existing shafts on the safety of operating trains

**DOI:** 10.1038/s41598-024-63622-7

**Published:** 2024-06-04

**Authors:** Changfu Huang, Shaohua Li, Zhenbo Zhang, Tiejun Yao, Xianming Shi, Jingwei Tian, Zhinan Hu, Yuhai Wang

**Affiliations:** 1 China Railway 15th Bureau Group Co.Ltd., Shanghai, 200070 China; 2 Shijiazhuang Tiedao University, Shijiazhuang, China; 3 China Railway Southwest Research Institute Co., Ltd., Chengdu, China; 4 China Academy of Railway Sciences Co., Ltd., Beijing, China

**Keywords:** Tunneling, Existing inclined shaft, Ventilation, Operating train, Transient pressure, Aerodynamic force, Engineering, Civil engineering

## Abstract

Given the influence of air intake from inclined shafts in existing tunnel ventilation systems on train comfort and aerodynamic safety, a numerical analysis method is used to study the comfort and aerodynamic safety of operating trains under three conditions—inclined shaft closed and inclined shaft open without and with air intake—and to explore the variation law of transient pressure and aerodynamic force (lift coefficient, transverse force coefficient, and overturning moment coefficient). Combined with practical engineering and requirements, the influence of inclined shaft air intake on train operation comfort and aerodynamic safety is analyzed. Through this research, the influence of using air intake from the inclined shaft of an existing tunnel, a ventilation scheme of the new Wushaoling Tunnel, on the comfort and aerodynamic force of trains is revealed, and the comfort and aerodynamic safety of trains in an actual project are evaluated, verifying the rationality of the ventilation scheme of the Wushaoling Tunnel.

## Introduction

To ensure the construction environment and progress during the construction period and the ventilation demand during the operation period, a ventilation inclined shaft is set up in a new tunnel. Sometimes, the ventilation inclined shaft of an existing tunnel can be used for air intake in the new adjacent tunnel, which undoubtedly improves work efficiency and reduces costs. However, using the ventilation inclined shaft of an existing tunnel to remove air will inevitably change the surrounding flow field when an operating train passes through the tunnel. In addition, a situation in which the air volume and wind speed are too high can greatly impact train operation safety and passenger comfort and even cause accidents.

Considering the safety of operating trains and the comfort of passengers crossing tunnels, many scholars have used numerical analysis methods to study the safety of operating trains in relation to the surrounding flow field. Xiang et al.^1^ calculated the aerodynamic coefficient of a high-speed train passing through a double-line simply supported box girder bridge and defined and determined the allowable value of the train operation overturning coefficient. Du et al.^2^ established a high-speed train eight-car formation dynamics model and a spatial fluctuating crosswind load model and explored the basic characteristics of train operation under fluctuating crosswinds and the safety limits of train speed and wind speed under these conditions. Luo et al.^3^ discussed the influence of embankment inclination change on the aerodynamic characteristics of high-speed trains during crosswind operation. Xie et al.^4^ studied the influence of sand-carrying strong crosswinds on the aerodynamic characteristics of high-speed trains under different embankments and evaluated the relationships among the transverse force coefficient, lift coefficient, overturning moment and embankment inclination angle. Liu et al.^5^ used the five-mass model to study the critical overturning wind speed curve of a vehicle under crosswinds and the influence of different parameters on its overturning. Ezoji et al.^6^ determined the overturning critical wind speed of the ICE2 train model. Cheli et al.^7^ optimized the aerodynamics of the new AnsaldoBreda EMUV250 train in terms of crosswind behavior. Zhou et al.^8^ studied the aerodynamic characteristics of high-speed trains accelerated under crosswind conditions and evaluated the effects of three acceleration values on the aerodynamic force/bending moment coefficients and safety indicators. Montenegro et al.^9^ proposed a discrete gusting method considering variable turbulence intensity and terrain type for the applicability of train operation safety evaluation on bridge decks at different heights. Niu et al.^10^ analyzed the influence of the coupling region on the aerodynamic performance of two multiunit trains entering a tunnel and passing each other in the tunnel. Yu et al.^11^ studied the influence of random wind with longitudinal and transverse components on high-speed trains and obtained the characteristic wind curve of high-speed trains and its diffusion range. Zhuang et al.^12^ studied the yaw effect of high-speed train side flow at two typical yaw angles, 30° and 60°, and analyzed the time-averaged flow pattern, turbulence statistics and surface force on the cross section of this train. Proppe et al.^13^ studied the problem of crosswind stability by using a stochastic model considering uncertainties. A wind model with nonstationary wind turbulence was established to analyze the crosswind stability of rail vehicles on straight and curved tracks. Huang^14^ and Morden^15^ combined this method with a model test. The former studied the transient aerodynamic characteristics of high-speed vehicles with body roll motion under crosswind conditions, and the latter verified the method for evaluating the aerodynamics of moving trains. Li^16^ proposed a numerical calculation method for the interaction between a high-speed train and airflow based on the coupling dynamics of vehicles and rails and aerodynamics.

Several scholars have also used model test methods to study the safety of operating trains relative to the surrounding flow field. Zhang et al.^17^ studied the effects of inclined tunnel entrances on the aerodynamics of trains and tunnels and revealed the physical mechanism through which inclined tunnel entrances slow initial compression waves. Niu et al.^18^ studied the effect of the Reynolds number on the aerodynamic force and pressure of trains at yaw angles of 0° and 15° and analyzed the difference in the Reynolds number effect between the front and rear trains. Gao et al.^19^ conducted a full-scale test on the pressure of high-speed streamlined trains under crosswinds, evaluated the influence of different crosswinds on the load, and analyzed the mean value and fluctuation law of the transverse force coefficient, lift coefficient and roll moment coefficient under different yaw angles. Liu et al.^20^ established a crosswind model based on the measured wind speed curve, studied the impact of single and continuous wind speed changes on train dynamics, performed parameter analysis of the wind speed change rate, maximum wind speed duration and amplitude wind speed changes, and proposed an estimation method for the wind speed range for safe train operation under sudden changes in wind speed. Bell et al.^21^ proposed a wind tunnel method for slipstream assessment and analyzed the influence of simulated ballast and track or flat ground on the wake structure. Gilbert et al.^22^ determined the basic changes in air movement caused by trains through instrument walls and box tunnels with 1/25 scale high-speed trains and evaluated the effects of constraints on the boundary layer and wake velocity. Tian^23^ systematically summarized the aerodynamic studies related to China's high-speed railway network. The progress of research on train aerodynamic drag reduction technology, train aerodynamic noise reduction technology, train ventilation technology, train crossing aerodynamics, train/tunnel aerodynamics, train/climate environment aerodynamics, and train/human aerodynamics is comprehensively discussed. Allain^24^ introduced the research status of railway-side air dynamics at home and abroad, provided European regulations and aerodynamic processes, and summarized some problems in the current research.

Through the above research, we see that the changes in the surrounding flow field have been fully studied in relation to train operation safety. However, a newly constructed tunnel removes air from the inclined shaft of an existing tunnel, and the influence of the change in the surrounding flow field on the safety of operating trains passing through the tunnel needs further study. On this basis, by relying on a new tunnel project near an existing operating tunnel, the change laws of the transient pressure and aerodynamic force (lift coefficient, transverse force coefficient and overturning moment coefficient) of a train were studied. These change laws of the train were analyzed under various conditions, and the influence of air extraction from the inclined shaft on the comfort and safety of train operation was determined. This research provides a basis for the subsequent use of existing tunnel ventilation inclined shafts in similar air projects.

## Problem posing

The new Wushaoling Tunnel of the Lan-Zhang-Zhang 3rd–4th-Line Railway is located on the east side of the existing Lan-Wu 2nd-Line Wushaoling Extralong Tunnel (existing tunnel), and the distance from the existing right-line tunnel is approximately 213 m (Zhangye end)–576 m (Lanzhou end). The length of the new Wushaoling Tunnel is 17,125 m, with a two-track tunnel with a designed speed of 250 km/h, a maximum burial depth of 952 m, and an altitude of 2700–3000 m at each tunnel entrance.

**Figure. 1:**
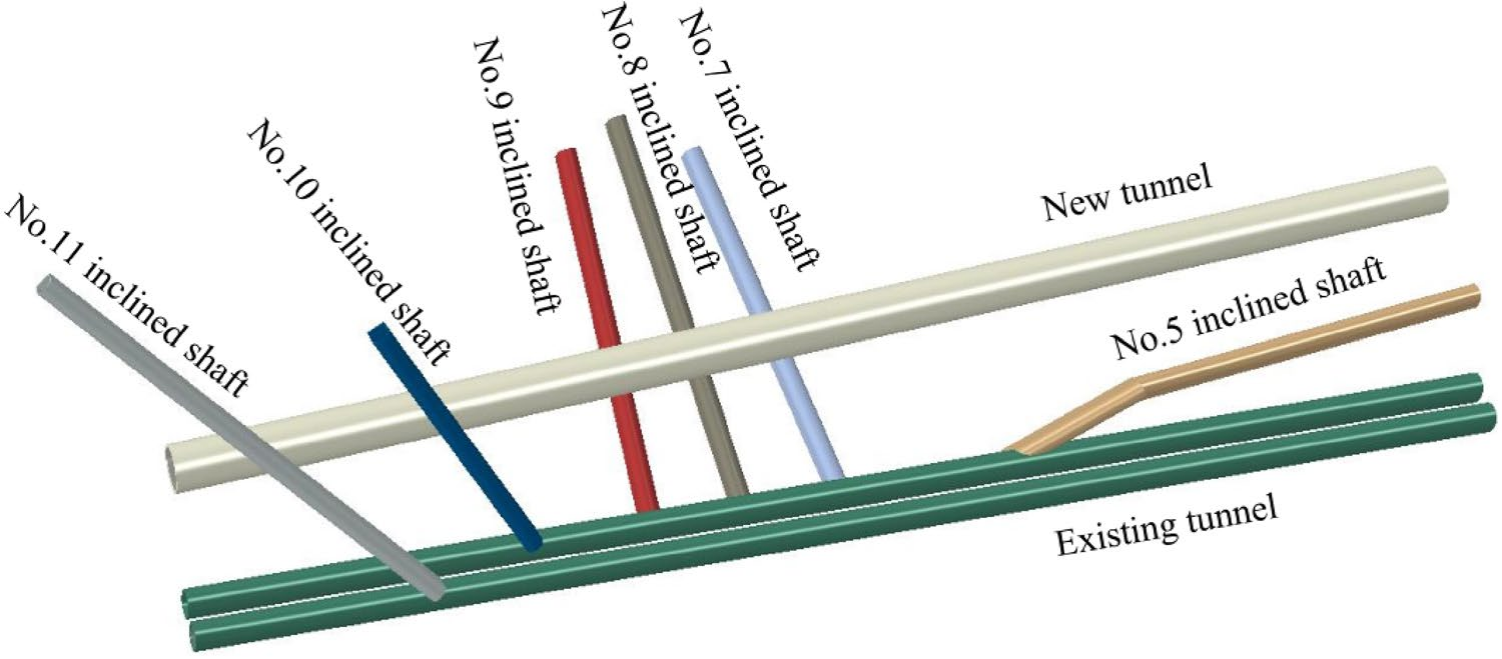
Three-dimensional relationship diagram of existing and new Wushaoling Tunnels and existing inclined shafts

Because many inclined shafts are established during the construction of existing tunnels, the inclined shafts of existing tunnels can be used for ventilation while the new Wushaoling Tunnel is constructed. Six inclined shafts are set up in the Xinwushaoling Tunnel, five of which are built on the basis of the Nos. 5, 7, 8, 9 and 10 inclined shafts of the existing tunnel. The new tunnel passes through the Nos. 7, 8 and 9 inclined shafts of the existing tunnel, respectively, and passes through the Nos. 10 and 11 inclined shafts of the existing tunnel. There is no cross relationship with the existing No. 5 inclined shaft. During the construction process, the Nos. 5, 7, 8 and 9 inclined shafts connect the right tunnel. The relative positions of the inclined shaft and the existing tunnel are shown in Fig. 1.

During the construction of a new tunnel, considering the spatiotemporal differences between the inclined shaft of some existing tunnels and the outside atmosphere, the connection between each branch hole of the inclined shaft and the inclined shaft of the existing line, and even the changes in the airflow direction and flow caused by the ventilation of the new tunnel through the auxiliary adit and the existing tunnel, the existing tunnel will experience various flow fields at different stages according to the construction progress. These flow fields will inevitably have an aerodynamic effect on the transient pressure and aerodynamic force of a train running in them. Therefore, it is urgent to study the dynamic influence of the flow field change in an existing long tunnel caused by the construction ventilation of adjacent tunnels on the aerodynamic effect index of trains.

## Evaluation standards for the comfort and safety of operating trains

### Transient pressure standard

The maximum transient pressure is given according to the relevant requirements of the Supplementary Regulations on Relevant Standards for Railway Tunnel Design and Construction (Railway Construction [2007] No. 88), as shown in Table 1 below.

**Table 1:** Maximum transient pressure requirements

Condition	Tunnel type	Maximum transient pressure requirements
The proportion of tunnels in the line is less than 10%, and less than 4 tunnels are passed every hour	Single hole single track tunnel	2 kPa/3 s
	Single hole double track tunnel	3 kPa/3 s
The proportion of tunnels in the line is greater than 25% or more than 4 tunnels are passed every hour	Single hole single track tunnel	0.8 kPa/3 s
	Single hole double track tunnel	1.25 kPa/3 s

### Aerodynamic standards for trains

When the inclined shaft takes air, it will produce a transverse force and lift force on the moving train, and the two forces will produce an overturning moment for the train. When the moment is too large, train overturning accidents may occur. According to the requirements of the Code for Evaluation and Test Identification of Railway Vehicle Dynamic Performance (GB5599-1985), the overturning coefficient D can be used to evaluate the aerodynamic safety of trains, and this coefficient needs to be no more than 0.8.

The expression for the overturning coefficient obtained according to the static moment balance is as follows:

1 $$D = \frac{{h_{g}^{\prime } }}{b}\left( {1 - \frac{\mu }{1 + \mu }} \right)\frac{{j_{a} }}{g} + \frac{{h_{w}^{\prime } p_{2w} LH}}{Pb}$$

where *h*_*g*_*'* is the total weight of the train converted to the height of the rail surface,

2 $$h_{g}^{\prime } = h_{g} + \frac{1}{1 + \mu }C_{y} P_{2}$$

*h*_*g*_ is the total weight center of the train converted to the height of the rail surface,

3 $$h_{g} = \frac{{P_{2} h_{g} + P_{1} r_{0} }}{P} = \frac{{h_{c} + \mu r_{0} }}{1 + \mu }$$

and *C*_*y*_ is the lateral displacement of the center of gravity of the vehicle body caused by the unit lateral force.

4 $$C_{y} = \frac{1}{{2K_{y} }} + h_{0} C_{y\phi }$$

In addition, *C*_*yφ*_ is the lateral displacement of the center of gravity of the vehicle body caused by the unit torque:

5 $$C_{y\phi } = \frac{{h_{0} }}{{2b_{2}^{2} K}}$$

*μ* is the set coefficient,

6 $$\mu = \frac{{P_{1} }}{{P_{2} }}$$

and *h*_*w*_*'* is the transverse force center of the car body converted to the height of the rail surface,

7 $$h_{w}^{\prime } = h_{w} + \left( {C_{y} - C_{y\phi } e} \right)P_{2}$$

In these formulas, *b* is half of the wheelbase of the wheel rail, the unit is mm; *r*_0_ is the height of the center of gravity of the bogie, the unit is mm; *j*_*a*_ is the transverse vibration acceleration of the carriage, the unit is m/s2; g is the gravitational acceleration, the unit is m/s2; *p*_2*w*_ is the pressure per unit area on the side of the carriage, the unit is kN; *L* is the length of the carriage, the unit is m; *H* is the height of the carriage, the unit is m; *P*_1_ is the mass of the bogie, the unit is kg; *P*_2_ is the weight of the carriage, the unit is kg; *h*_*c*_ is the height from the center of gravity to the rail surface, the unit is mm; *h*_0_ is the height from the center of gravity to the centerline of the axle, the unit is mm; *b*_2_ is half of the transverse span of the second series spring, the unit is mm; *K*_*y*_ is the transverse stiffness of a bogie spring, the unit is kN/mm; *K* is the vertical stiffness of a bogie spring, the unit is kN/mm; *h*_*w*_ is the transverse force center of the carriage to the rail surface height, the unit is mm; and *e* is the distance between the center of transverse force and the center of gravity of the carriage, the unit is mm.

According to Formulas (1)–(7), the transverse force and lift force generated by the inclined shaft cause the train to overturn. Therefore, the train aerodynamic standard is characterized by the train overturning coefficient.

## Model building

In this paper, the numerical analysis method is used to analyze the influence of inclined shaft air intake on trains running in existing operating tunnels.

### Rationality verification of the research methods

**Figure. 2:**
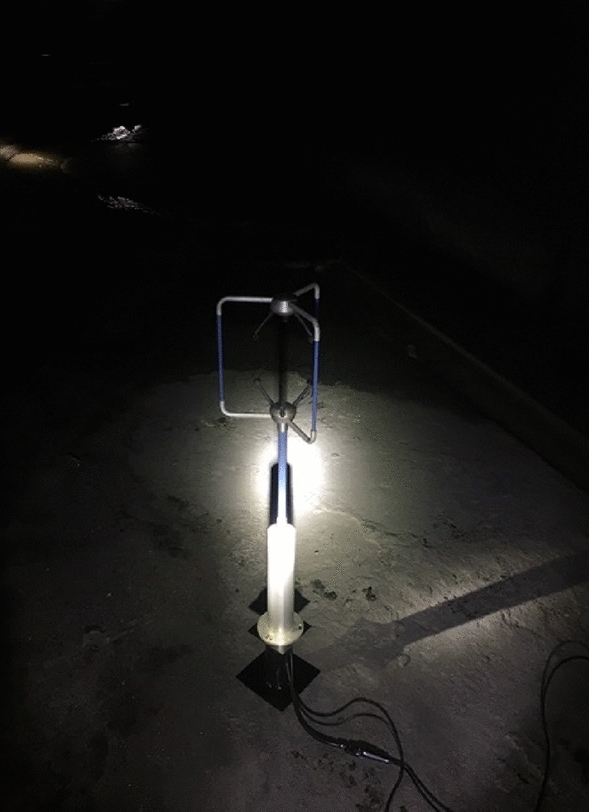
On-site wind speed test

In this paper, the numerical simulation method is used to investigate the influence of air extraction from inclined shaft on trains running in existing operating tunnels. Field test of wind speed was carried out on the left line of the existing Wushaoling tunnel. In the test, British GILL’s three-dimensional ultrasonic wind speed and direction instrument WindMaster (see Fig. 2) was used to test wind speed. This instrument can accurately measure the wind speed in the range of 0–50 m/s, and can provide vertical, normal and tangential wind speed data in three directions, and can provide a data output rate of 20 Hz. The measuring instrument was fixed by the instrument frame, and the probe pad was raised to 2 m from the ground. The three-direction wind speed was collected, and the parameters in the numerical simulation were modified by comparing and analyzing the results with the numerical simulation results (see Table 2).

**Table 2:** Comparison of wind speed data at measuring points

Wind speed of measuring point	Field measured results (m/s)	Numerical calculation results (m/s)	Deviation (%)
Maximum value	23.11	22.38	− 3.2
Minimum value	− 7.29	− 6.97	− 4.4

As shown in Table 2, the differences between the maximum and minimum longitudinal wind speeds of the tunnel are 3.2% and 4.4%, respectively, and the deviation is less than 5%. Therefore, the research method adopted in this paper can reasonably predict the actual situation in the field and can be used for subsequent research and analysis.

### Finite element model

#### Training model

This section takes the CRH380A EMU, commonly used in high-speed railways, as the research object. The eight-car train is 200 m in length, 3.38 m in width and 3.9 m in height, with a cross-sectional area of 11.2 m^2^ and a speed of 350 km/h. To facilitate calculation, the training model is simplified:

1. When establishing the model, the train body is simplified into a series of smooth surfaces, and the roughness of the outer surface of this body is considered.
2. The peripheral and bottom components of the car body are simplified, and the detailed structures of the bogie, air conditioner and pantograph are ignored. A graphic design form is chosen through the skirt plate under the car to connect with it so that a narrow gap is formed between the car and the ground, and the fluid movement in the gap is used to simulate the movement of the flow field between the car and the ground.

#### Tunnel model

**Figure. 3:**
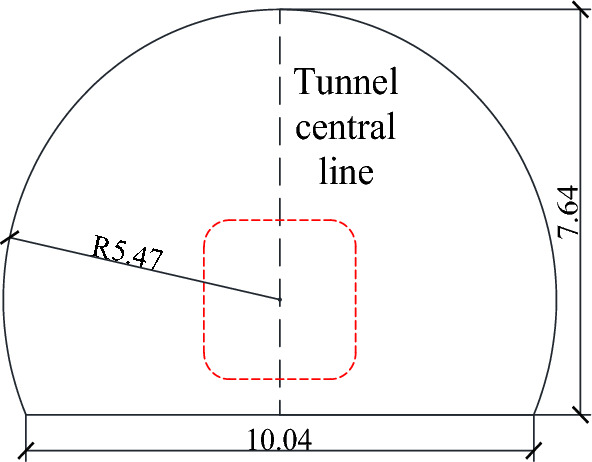
Section view of the existing tunnel

**Figure. 4:**
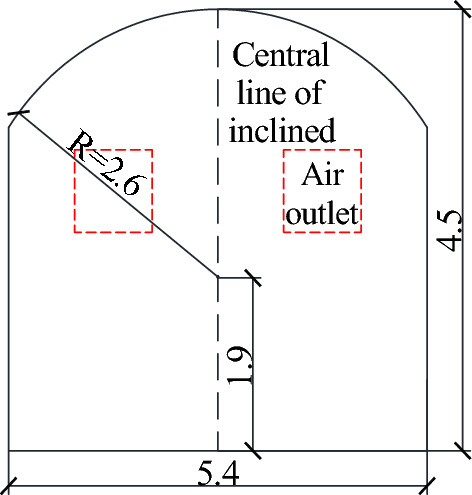
Inclined shaft section diagram (including the location of air intake)

The existing tunnel is set according to the standard section of a single line applicable to 350 km/h, and the tunnel section is shown in Fig. 3. This section has a headroom area of 70 m^2^ and a total tunnel length of 1000 m. The inclined shaft is vertically arranged in the middle of the tunnel with a length of 100 m. A protective door is arranged at a 1 m distance between the inclined shaft and the junction of the existing tunnel. When the protective door is closed, the flow field inside the inclined shaft can be completely blocked. The other end of the inclined shaft is connected to the construction tunnel, the section of the construction tunnel is the same as that of the existing tunnel, the sealing wall is arranged 20.5 m from the interface between the inclined shaft and the construction tunnel, and only two air intake outlets with a length and width of 1.5 m are retained (Fig. 4).

#### Computational region

**Figure. 5:**
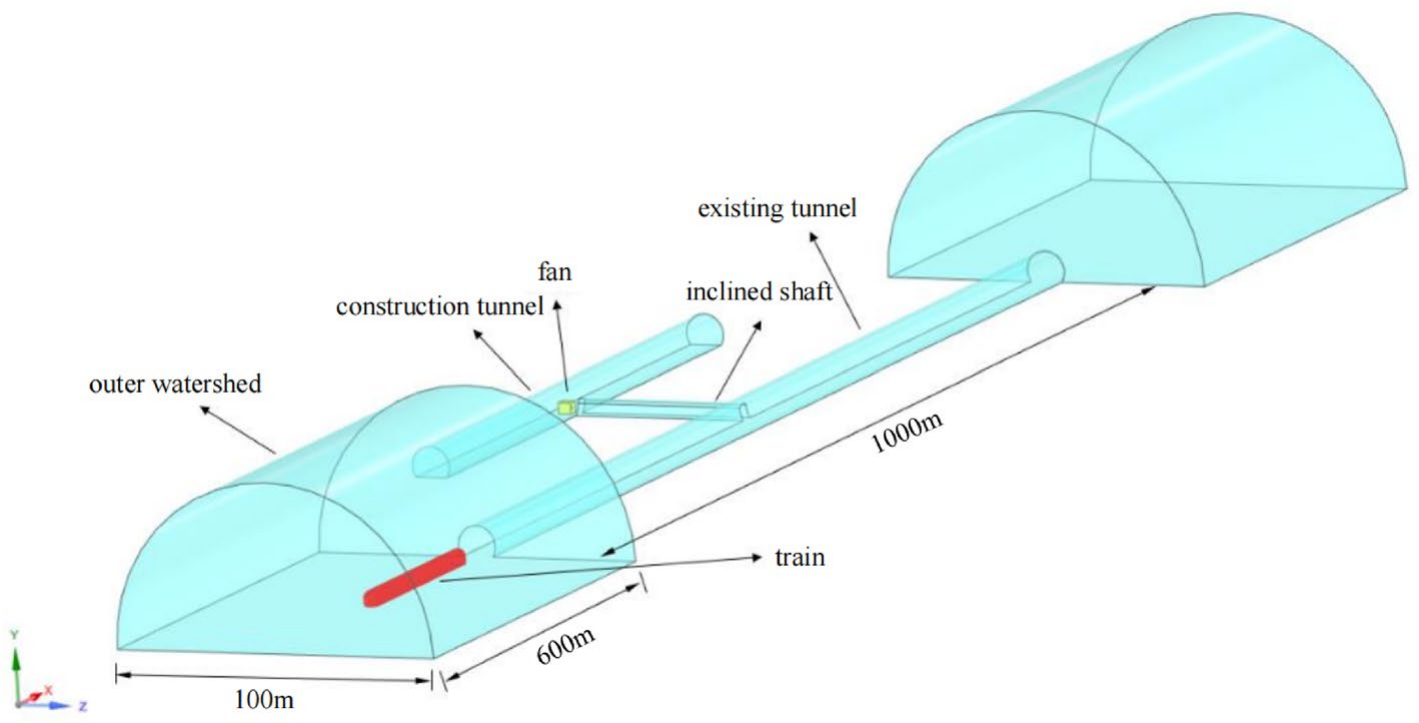
Calculation domain diagram

The calculation domain in the numerical simulation includes the tunnel domain and two outer domains. The two outer domains have the same semi-cylindrical body, the bottom surface has a radius tenfold the span of the existing tunnel and a length of 100 m, the length of the outer domain is 600 m, and the train is placed 60 m from the tunnel entrance. The tunnel domain includes existing tunnels, construction tunnels and inclined shafts. The existing tunnel is 1000 m long, the inclined shaft is 100 m long, and the construction tunnel is 500 m long. The coordinate system specified in this paper is shown in Fig. 5. The train's running direction is the x-axis forward, the y-axis forward points to the top of the tunnel, and the z-axis follows the right-hand screw rule.

#### Boundary conditions

The whole calculation domain is divided into a static part and a sliding part, and the common surface of these parts is set as the interface boundary to realize the relative motion between the train and the tunnel. The pressure inlet and pressure outlet boundaries are adopted for the outer domains at both ends of the static part. The wall and bottom surface of the existing tunnel, construction tunnel and inclined shaft are set as a nonslip wall boundary; the two tuyeres of the inclined shaft sealing wall are set as a velocity inlet boundary, and the wind speed is 5 m/s; the palm surface of the construction tunnel is set as a nonslip wall boundary; the other end is set as a pressure outlet boundary; the bottom surface of the sliding part and the train surface are set as a nonsliding wall boundary; and the two ends of the surface are set as a pressure inlet and pressure outlet boundary. The roughness heights of the tunnel, inclined shaft wall and train surface are 0.02 m and 0.018 m, respectively. The medium of the flow field in the tunnel is set as a compressible ideal gas, and the turbulence model is the *k*−*ε* model.

#### Mesh subdivision

**Figure. 6:**
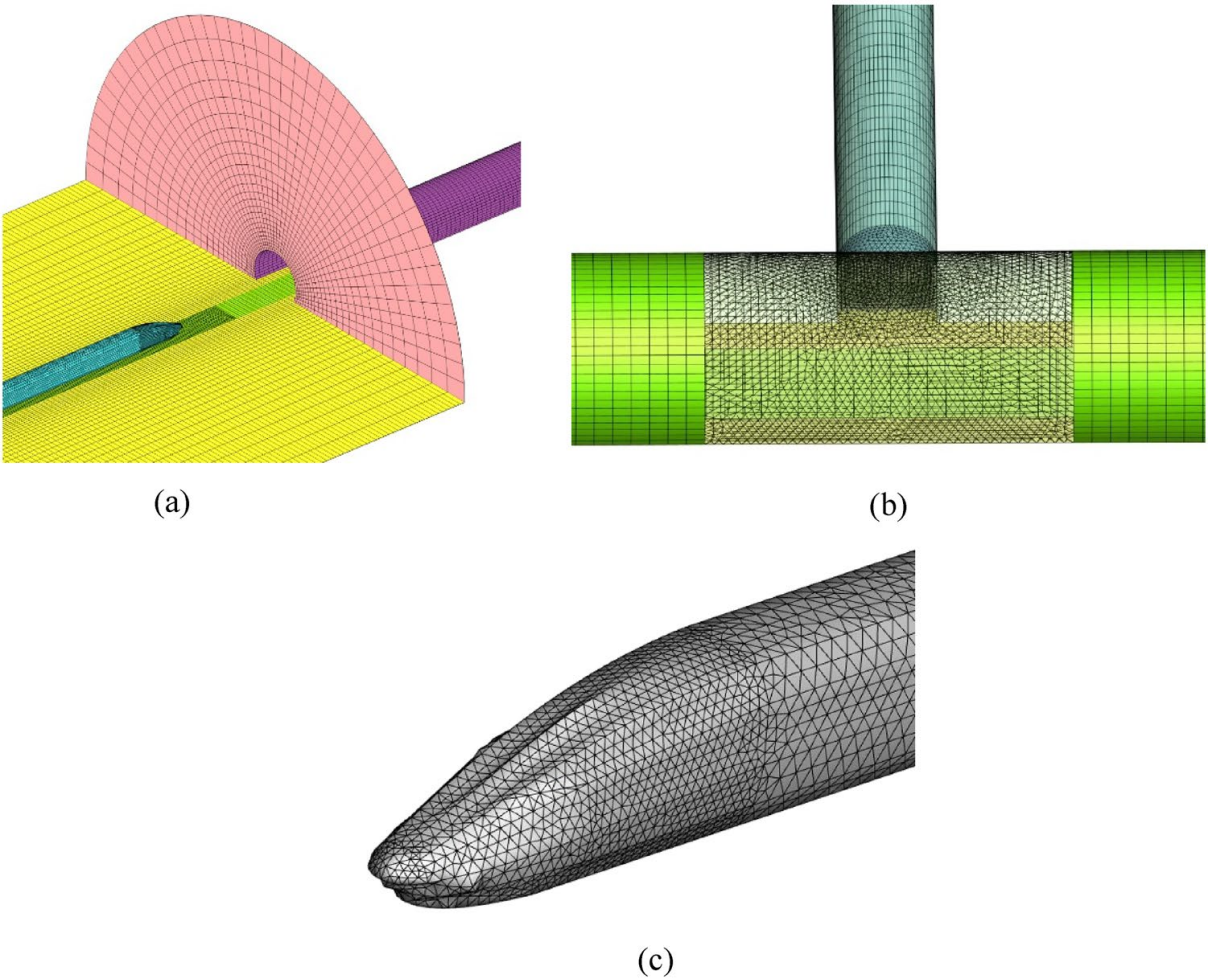
Mesh subdivision

The calculation domain is divided by an unstructured hexahedral mesh with a minimum mesh size of 0.1 m. The inclined well grid was encrypted, and approximately 33 million hexahedral units were calculated after the model was dispersed (Fig. 6).

### Calculation conditions and arrangement of measuring points

#### Calculation conditions

The three conditions of inclined shaft closed (Condition A), inclined shaft open without air intake (Condition B) and inclined shaft open with air intake (Condition C) are calculated. Based on the ideal ventilation demand, the air intake of the inclined shaft is 10,800 m^3^/min. The specific calculation conditions are shown in Table 3.

**Table 3:** Calculation conditions

Sign	Condition	Remark
A	Inclined shaft closing	The closure of the protective door causes the flow field in the existing tunnel to not communicate with the inclined shaft
B	Inclined shaft opening without air intake	
C	Inclined shaft opening with air intake	The air intake of inclined shaft is 10,800 m^3^/min

#### Monitoring arrangement

In train operation, the aerodynamic force mainly includes the lift force, transverse force, overturning moment and air resistance. To conduct quantitative research, the air resistance coefficient *C*_Fx_, lift coefficient *C*_Fy_, transverse force coefficient *C*_Fz_ and overturning moment coefficient *C*_Mx_ of each carriage are defined in the numerical simulation software, and their relationships with the air resistance *F*_x_, lift force *F*_y_, transverse force *F*_z_ and overturning moment *M*_x_ are as follows:

8 $$C_{Fx} = \frac{{F_{x} }}{{(1/2)\rho v^{2} S_{0} }}$$

9 $$C_{Fy} = \frac{{F_{y} }}{{\left( {1/2} \right)\rho v^{2} S_{y} }}$$

10 $$C_{Fz} = \frac{{F_{z} }}{{\left( {1/2} \right)\rho v^{2} S}}$$

11 $$C_{Mx} = \frac{{M_{x} }}{{\left( {1/2} \right)\rho v^{2} Sh}}$$

where *ρ* is the air density and *ρ* = 1.225 kg/m^3^ is taken; *v* is the airflow velocity, m/s; *S*_0_ is the maximum cross-sectional area of the train, which is 11.6 m^2^; *S*_y_ is the overlooked projected area of the carriage, 85.8 m^2^ (Fig. 5 xz plane in the coordinate system); *S* is the projected area of the side view of the carriage, 99.0 m^2^; and *h* is the height of the carriage, 3.9 m.

**Figure. 7:**
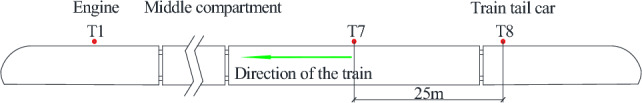
Schematic diagram of train measuring point layout

Pressure measuring points are arranged in the middle of the top surface of each carriage, numbered T1–T8 from the beginning to the end (see Fig. 7), and the pressure changes at the monitoring measuring points can be extracted.

**Figure. 8:**
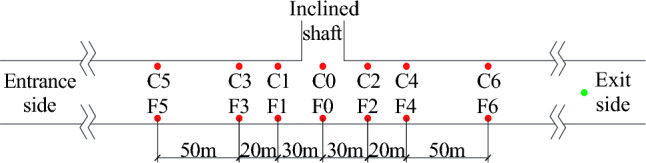
Schematic diagram of test points in the tunnel

Taking the center of the inclined shaft as the reference point, pressure measuring points are arranged on the tunnel wall near the inclined shaft at intervals of 30 m, 50 m and 100 m along the longitudinal sides of the tunnel, and the same number of pressure measuring points are arranged symmetrically along the midline of the tunnel away from the inclined shaft. Longitudinally, the front of the inclined shaft is near the entrance, and the rear of the inclined shaft is on the other side. The height of the measuring point is 2.75 m. A layout diagram of the test points in the tunnel is shown in Fig. 8.

## Calculation results

### Transient train pressure

According to the calculation results, the surface pressure at the first train measuring point (T1), the middle train measuring point (T4) and the last train measuring point (T8) is extracted. The calculation results are shown in Table 4.

**Table 4:** Train surface pressure

Item	T1			T4			T8		
	Condition A	Condition B	Condition C	Condition A	Condition B	Condition C	Condition A	Condition B	Condition C
Maximum positive pressure (Pa)	1944.1	1798.6	1783.8	911.1	904.5	893.7	160.3	225.8	224.9
Maximum negative pressure (Pa)	− 4424.9	− 3408.7	− 3367.7	− 5170.4	− 4118.8	− 4060.1	− 5525.5	− 4672.5	− 4698.8

As shown in Table 4, when the train passes through the tunnel, the negative pressure on the surface of the car body is greater than the positive pressure. When the screen door is opened, due to the “wave separation” effect of the inclined shaft, the maximum negative pressure at the measuring point is obviously lower than that when the inclined shaft is closed, but whether the inclined shaft takes air after the inclined shaft is opened has little influence on the mitigation effect of the negative pressure on the surface of the car body. Compared to those under the closed inclined shaft, the maximum negative pressures at the measuring points of the head car, middle car and tail car are reduced by 23.0%, 20.3% and 15.4%, respectively, when the inclined shaft is open and no air is taken and by 23.8%, 21.5% and 15.0%, respectively, when the inclined shaft is open and air is used.

**Figure. 9:**
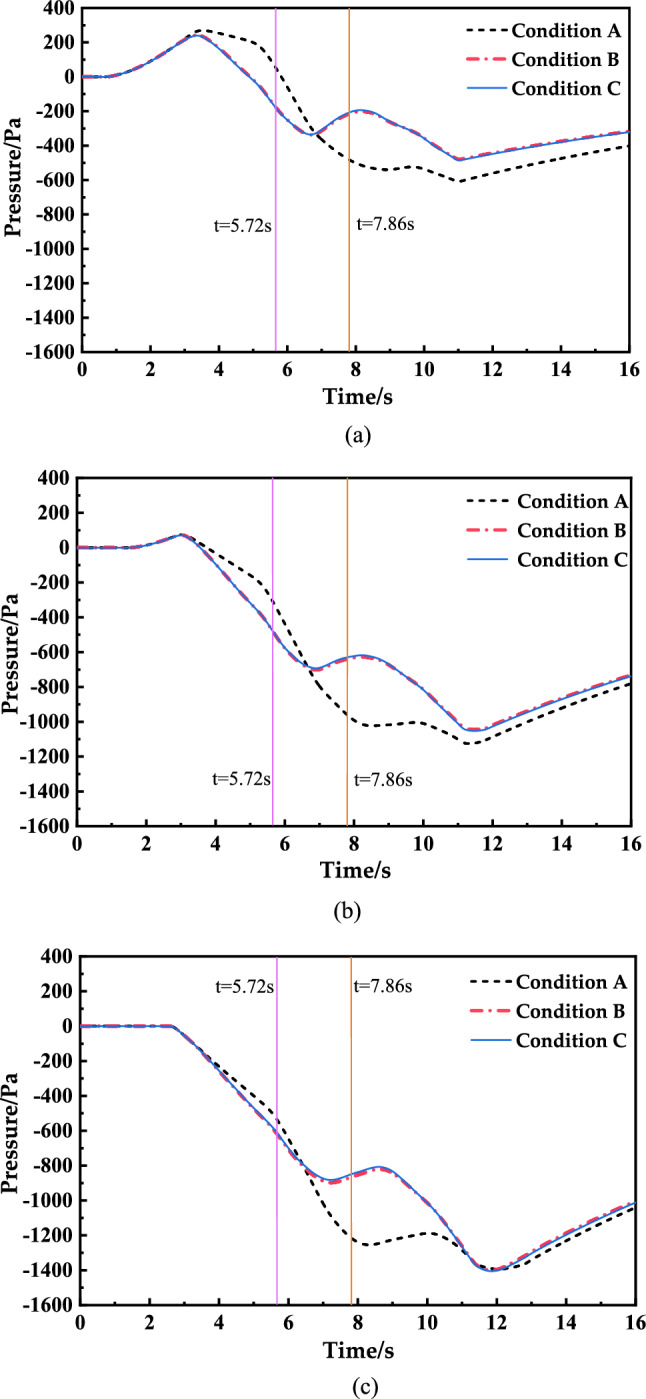
Transient pressure in the carriage

According to the standard, the factory seal index of the CRH380A EMU is at least 12 s. The transient pressure curves of the head car, middle car and tail car with a dynamic sealing index of 12 s were extracted, as shown in Fig. 9, The time for the car head to reach the front edge of the inclined shaft is 5.72 s; the time for the tail of the car to leave the rear edge of the inclined shaft is 7.86 s.

As shown in Fig. 8, the corresponding curves for Conditions B and C basically coincide, so whether the inclined shaft takes air slightly influences the transient pressure in each carriage. The maximum variation amplitudes of the 3 s transient pressure in the head car, middle car and rear car under different conditions are extracted from Fig. 8, as shown in Table 5 below.

**Table 5:** 3s transient pressure in the car

Item	Head car			Middle car			Tail car		
	Condition A	Condition B	Condition C	Condition A	Condition B	Condition C	Condition A	Condition B	Condition C
Maximum value (Pa/3 s)	701.3	565.3	560.8	625.5	516.1	510.8	678.1	538.4	530.1

As shown in Table 5, the presence of an inclined well can reduce the pressure variation amplitude within 3 s of the carriage, and the variation amplitude further decreases when the inclined well is in air, which is more comfortable for train operation than the other two operating conditions. However, whether the inclined well takes air has no obvious effect on the transient pressure change inside the carriage. In summary, air extraction from inclined shafts has little effect on train comfort.

### Aerodynamic force

#### Lift coefficient

**Figure. 10:**
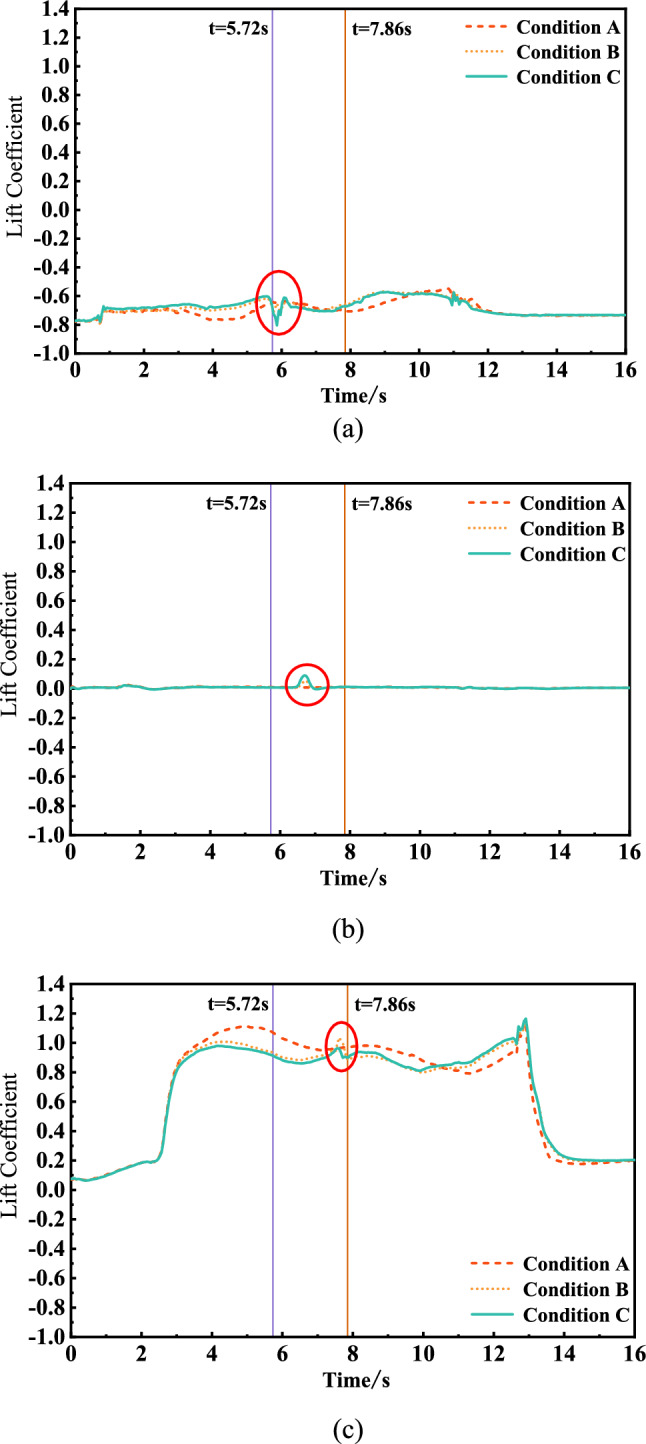
Time history curve of lift coefficient

The time‒history curves of the lift coefficients of the head, middle and tail trains passing through the tunnel under the three working conditions were calculated, as shown in Fig. 10. The maximum and minimum lift coefficients of the trains at different positions under the three working conditions were obtained according to Fig. 10, as shown in Table 6. The negative values in Fig. 10 and Table 6 indicate that the train forces are directed toward the track.

**Table 6:** Lift coefficient

Lift coefficient	Head car			Middle car			Tail car		
	Condition A	Condition B	Condition C	Condition A	Condition B	Condition C	Condition A	Condition B	Condition C
Maximum value	− 0.55	− 0.56	− 0.57	0.02	0.05	0.09	1.15	1.16	1.17
Minimum value	− 0.80	− 0.80	− 0.80	− 0.02	− 0.02	− 0.02	0.07	0.07	0.06

As shown in Fig. 10, when the train passes through the tunnel, the head car is always pressed toward the track, and the tail car is lifted upward by force after entering the tunnel. According to the calculation results shown in Fig. 10 and Table 6, neither the maximum nor the minimum lift coefficient of the front and rear cars differs considerably under the three working conditions. However, when the inclined shaft is closed, the lift coefficient of the middle vehicle passing through the inclined shaft section is − 0.02 to 0.02, and when the inclined shaft is opened without wind and with wind, it is − 0.02 to 0.05 and − 0.02 to 0.09, respectively. A tendency to uplift occurs briefly, and this trend becomes more obvious when the wind is used. In addition, after the inclined shaft is opened, when the first car reaches the inclined shaft, the force on the side pointing to the track suddenly increases and subsequently decreases, exhibiting a "nodding" trend. When the tail car moves from the inclined shaft, the lift force first increases and subsequently decreases within a short time, and a "head up" trend occurs.

#### Transverse force coefficient

**Figure. 11:**
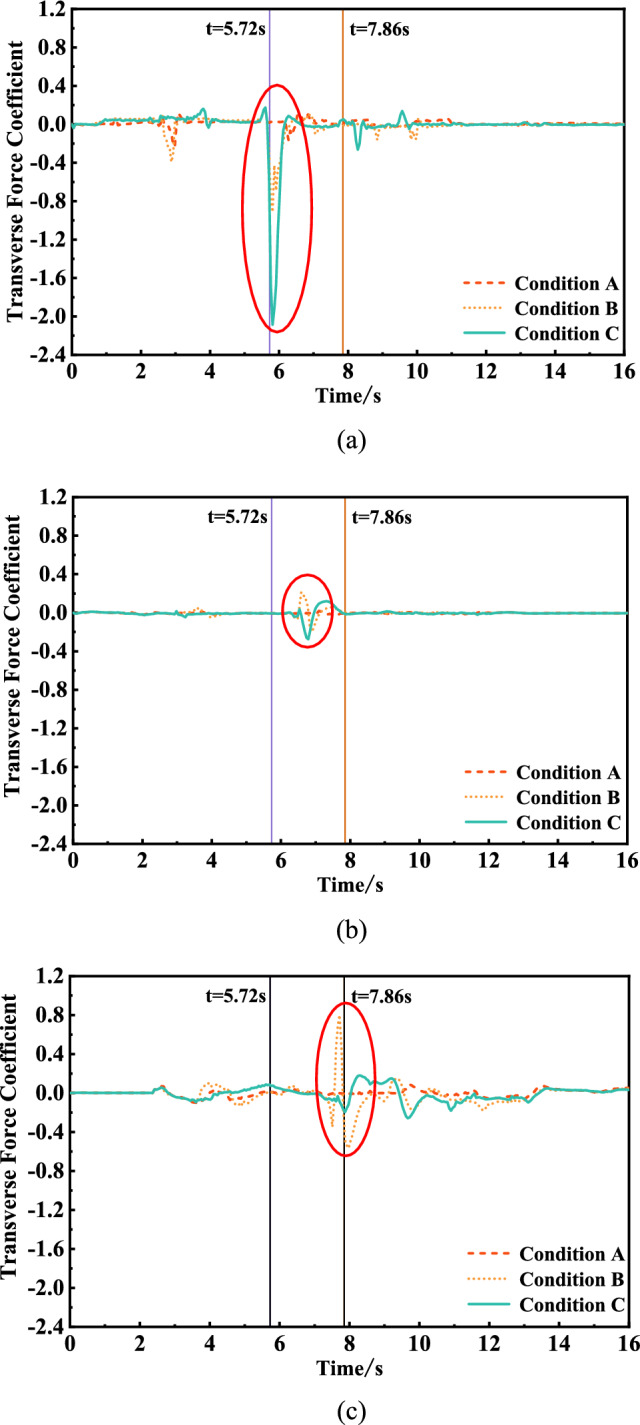
Time history curve of transverse force coefficient

The time‒history curves of the transverse force coefficients of the front, middle and rear trains passing through the tunnel under the three working conditions were calculated, as shown in Fig. 11. The maximum and minimum transverse force coefficients of the trains at different positions under the three working conditions were obtained according to Fig. 11, as shown in Table 7 below. The negative values of the data in Fig. 11 and Table 7 indicate that the force of the train points to the side of the inclined shaft.

**Table 7:** Maximum and minimum values of transverse force coefficient

Transverse force coefficient	Head car			Middle car			Tail car		
	Condition A	Condition B	Condition C	Condition A	Condition B	Condition C	Condition A	Condition B	Condition C
Maximum value	0.12	0.15	0.18	0.02	0.21	0.12	0.09	0.80	0.18
Minimum value	− 0.23	− 0.91	− 2.09	− 0.02	− 0.20	− 0.28	− 0.10	− 0.57	− 0.26

Figure 11 and Table 7 show that the transverse force coefficients of the three working conditions differ greatly when the head car arrives at the inclined shaft, the middle car passes through the inclined shaft and the tail car leaves the inclined shaft, and the transverse force on the train increases considerably when the inclined shaft is opened. After the inclined shaft is opened, when the train head car reaches the inclined shaft, the force on the side pointing to the inclined shaft increases sharply and then decreases rapidly. The maximum transverse force coefficient of the inclined shaft is − 0.91 and − 2.09 without and with taking air, respectively, increasing nearly 1.3-fold. When the middle car passes through the inclined shaft, the transverse force coefficient without taking air is − 0.20 to 0.21, and the force direction first deviates from and then points to the inclined shaft. When air is taken, the corresponding transverse force coefficient ranges from − 0.28 to 0.12. Compared with when no air is used, when the force on the inclined shaft increases, the force on the deviating inclined shaft decreases, but the difference is not substantial. The order of the force direction is opposite to that when air is not used. When the tail car is driven from the inclined shaft, the transverse force coefficient of the inclined shaft without air intake changes from 0.80 to − 0.57 in a short time, and the swing trend is obvious, while the transverse force coefficient of the inclined shaft with air intake changes from − 0.26 to 0.18, and the change is much more gradual.

Therefore, a train passing through the inclined shaft will suffer an increased transverse force and an increased swing trend. Compared with the condition without air intake, air intake from the inclined shaft increases the swing degree of the head car to the side of the inclined shaft, but it is conducive to the smooth operation of the tail car.

#### Overturning moment coefficient

**Figure. 12:**
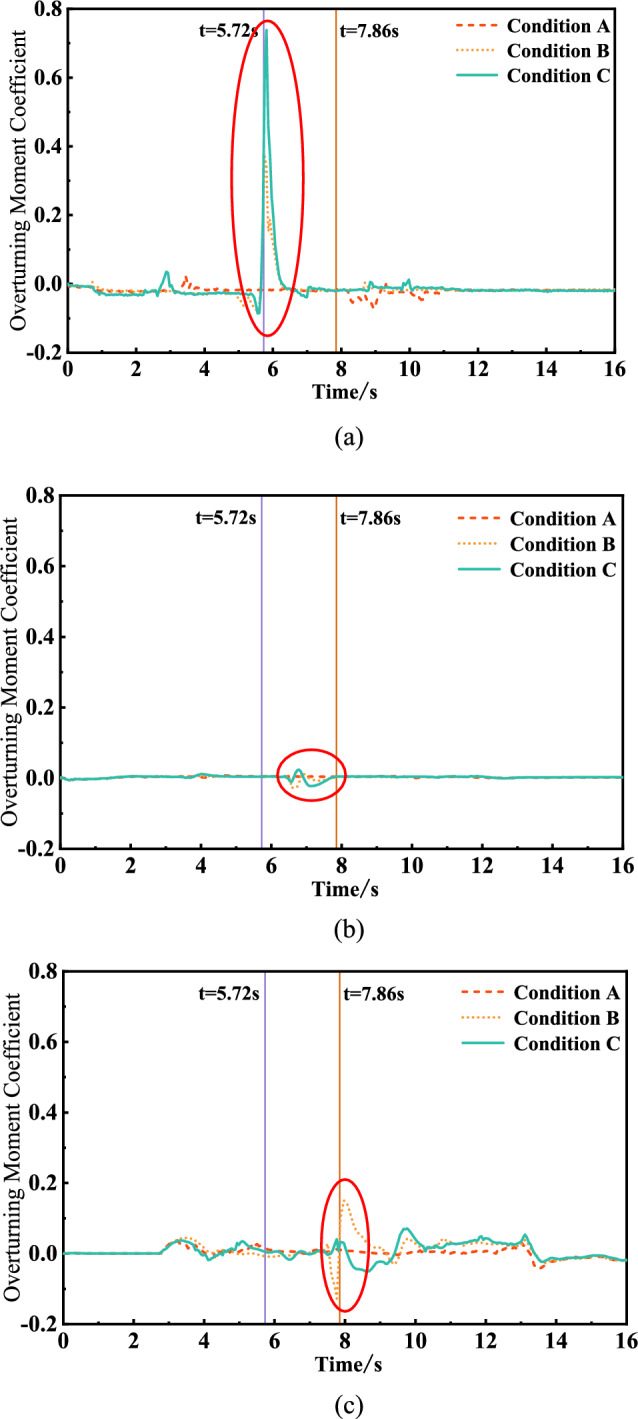
Time history curve of overturning moment coefficient

Figure 12 shows the calculated time‒history curves of the overturning moment coefficients of the head, midtrain and tail car while the train passed through the tunnel under the three working conditions. According to Fig. 12, the maximum and minimum lift coefficient values of trains at different positions under the three working conditions were obtained, as shown in Table 8. The positive values of the data in Fig. 12 and Table 8 indicate that the train tends to sway to the side of the inclined shaft.

**Table 8:** Overturning moment coefficient

Overturning moment coefficient	Head car			Middle car			Tail car		
	Condition A	Condition B	Condition C	Condition A	Condition B	Condition C	Condition A	Condition B	Condition C
Maximum value	0.02	0.37	0.74	0.01	0.01	0.02	0.04	0.15	0.07
Minimum value	− 0.07	− 0.09	− 0.09	− 0.01	− 0.03	− 0.02	− 0.11	− 0.13	− 0.11

Figure 12 and Table 8 show that the overturning moment coefficients of the three working conditions differ greatly between when the head car reaches the inclined shaft and the tail car leaves the inclined shaft, and the overturning moment of the train increases considerably when there is an inclined shaft.

**Figure. 13:**
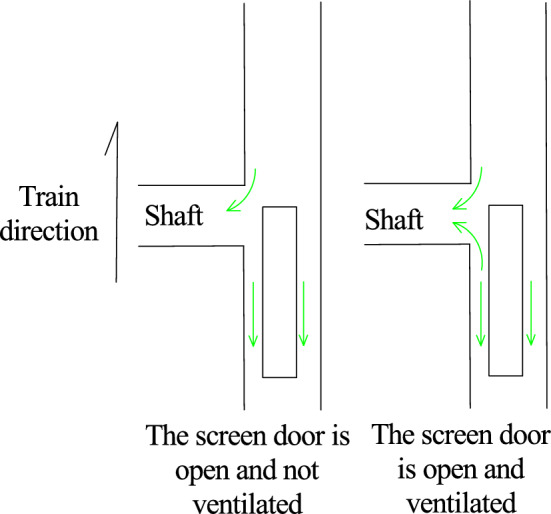
The gas flow field diagram when the head car passes through the inclined shaft under different working conditions

After the inclined shaft is opened, the overturning moment coefficient of the train head car suddenly increases greatly when it reaches the inclined shaft and then decreases rapidly. The maximum overturning moment coefficient of the inclined shaft is 0.37 when no air is taken and doubles to 0.74 when air is taken. This is because the aerodynamic force on the front of the vehicle will be reduced due to the ‘pressure relief’ of the inclined shaft when the front of the vehicle passes through the inclined shaft with the screen door open and no air intake. However, when the head of the vehicle passes through the inclined shaft where the screen door is opened and the wind is taken, the head of the vehicle will be affected by the wind force, resulting in an increase in the variation of the overturning moment coefficient of the head vehicle. The gas flow field diagram is shown in Fig. 13.

**Figure. 14:**
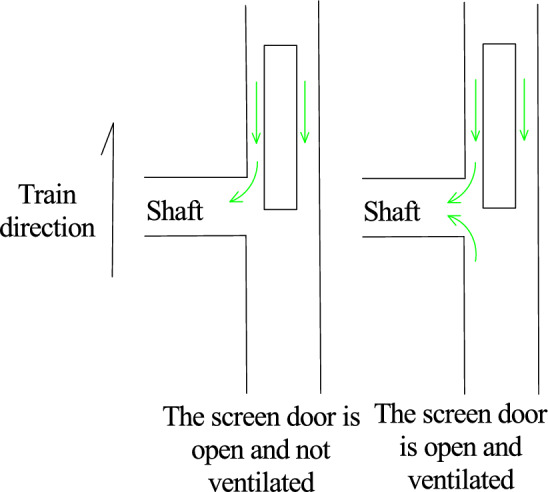
The gas flow field diagram when the tail car passes through the inclined shaft under different working conditions

When the tail car of the train leaves the inclined shaft, the overturning moment coefficient of the inclined shaft without air extraction promptly changes from − 0.13 to 0.15, while the overturning moment coefficient of the inclined shaft with air extraction varies from − 0.11 to 0.07 in a relatively gradual manner. Because the tail of the car is affected by the ' breaking wind ' of the front carriage when passing through the inclined shaft under the two conditions of no wind and wind, the change range is small. However, due to the air intake of the fan, the tail of the car is subjected to a smaller aerodynamic force to make its change smaller than that of the screen door open and no air intake. The gas flow field diagram is shown in Fig. 14.

**Figure. 15:**
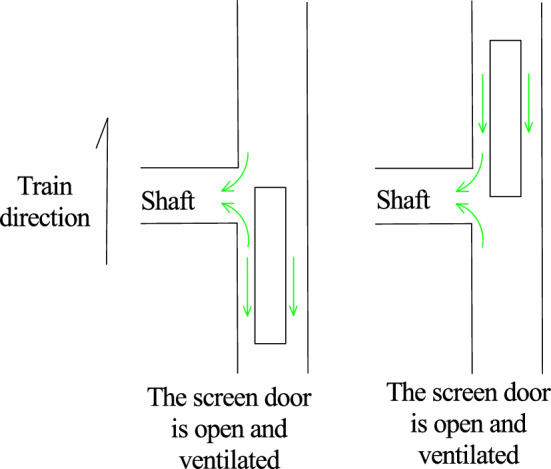
The gas flow field diagram of the front and rear of the vehicle passing through the inclined shaft under the working condition of the air inclined shaft

After the inclined shaft is opened and the wind is taken, the change range of the overturning moment coefficient of the head car is much larger than that of the tail car. When the head of the vehicle passes through the inclined shaft where the screen door is opened and the wind is taken, the head of the vehicle will be subjected to the wind and the aerodynamic force will mutate in the head of the vehicle, resulting in an increase in the variation of the lateral force coefficient and the overturning moment coefficient. When the rear of the vehicle passes through the inclined shaft where the screen door is opened and the wind is taken, the aerodynamic force does not change abruptly because of the 'breaking wind 'effect of the front compartment. The gas flow field is shown in Fig.15.

**Figure. 16:**
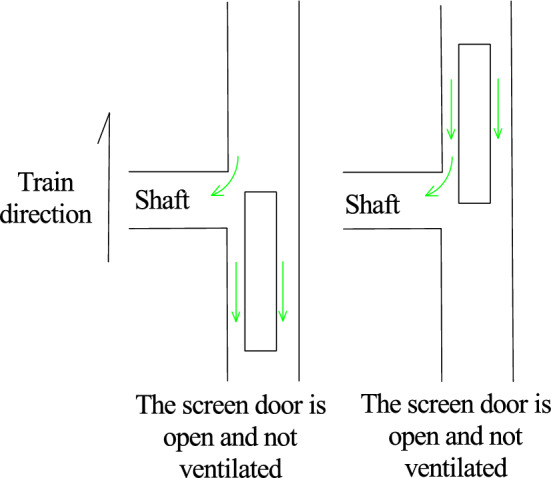
The gas flow field diagram of the front and rear of the vehicle passing through the inclined shaft under the condition of no wind inclined shaft

When the inclined shaft is opened and the wind is not taken, the variation range of the overturning moment coefficient of the head car is slightly larger than that of the tail car. When the head of the vehicle passes through the inclined shaft with the shield door open and no wind, the aerodynamic force will change suddenly in the head of the vehicle, but the sudden change of the aerodynamic force will be reduced due to the ' pressure relief ' of the inclined shaft. Compared with when the front of the car passes through the inclined shaft closed by the screen door, the change range is slightly increased. Because of the ' breaking wind ' effect of the front carriage, the variation of the rear of the two cases is equivalent. The gas flow field is shown in Fig. 16.

When the middle car passes through the inclined shaft, the overturning moment coefficients corresponding to the closed inclined shaft and the inclined shaft without and with air intake are − 0.01 to 0.01, − 0.03 to 0.01 and − 0.02 to 0.02, respectively. Therefore, the possibility of the train rolling increases when it passes through the inclined shaft. Compared the condition with no air intake, inclined shaft air intake increases the inclination degree of the head car toward the side of the inclined shaft but inhibits the inclination trend of the tail car.

## Engineering application

### Project overview

#### Project introduction

The new Wushaoling Tunnel takes air from ventilation inclined shafts Nos. 5, 7, 8, 9 and 10 of the existing tunnels. The planar relationship between the above inclined shafts and the existing tunnels is shown in Fig. 1.

**Figure. 17:**
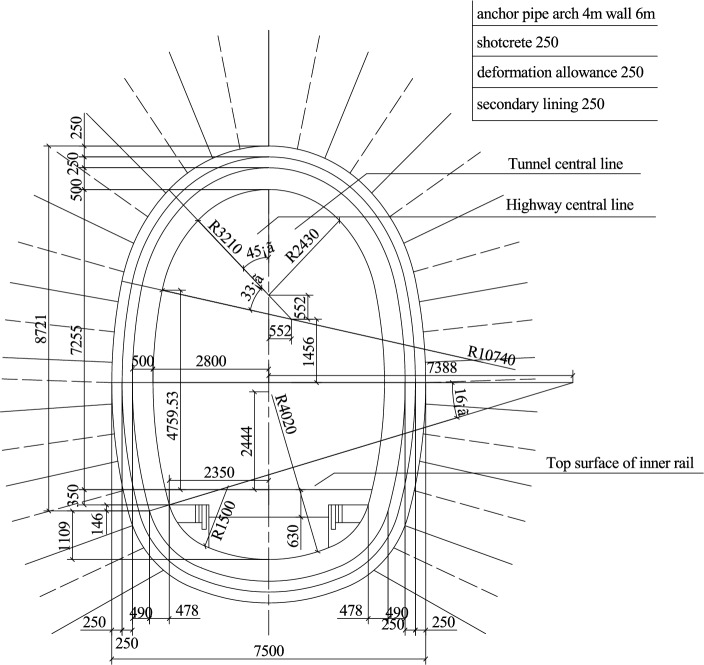
Tunnel profile of the right line of Wushaoling Mountain

The trains operating in the existing Wushaoling Tunnel are HXD1D type, with a height of 4.4 m, a width of 3.1 m, a length of 200 m (8 knots) and a speed of 120 km/h. The existing right-line tunnel of Wushaoling is 20050 m long and has a cross-sectional area of 30 m^2^, as shown in Fig. 17.

#### Ventilation scheme

**Figure. 18:**
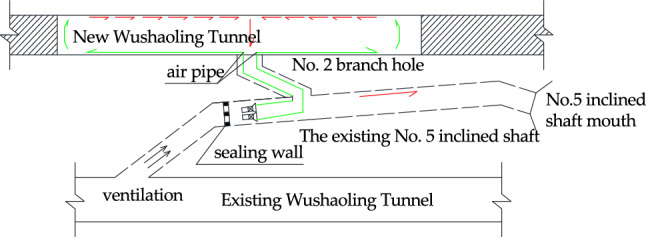
Schematic diagram of the relative position of inclined Shaft No. 5 and the new and existing Wushaoling tunnel

The inclined shafts have similar air extraction parameter settings. The air extraction situation of only the No. 5 inclined shaft is introduced here, and the other inclined wells are not described again. Existing inclined shaft No. 5 has a length of 2182.35 m, a comprehensive slope of 11%, an angle of 45° with respect to the existing railway, and a section area of 29.85 m^2^. The relative position diagram of the new and existing Wushaoling tunnels is shown in Fig. 18. The green arrow in the figure indicates that the gas is transported to the construction surface by the fan, and the red arrow indicates that the gas is discharged to the ground through the No. 2 branch hole and the No. 5 existing inclined shaft.

**Figure. 19:**
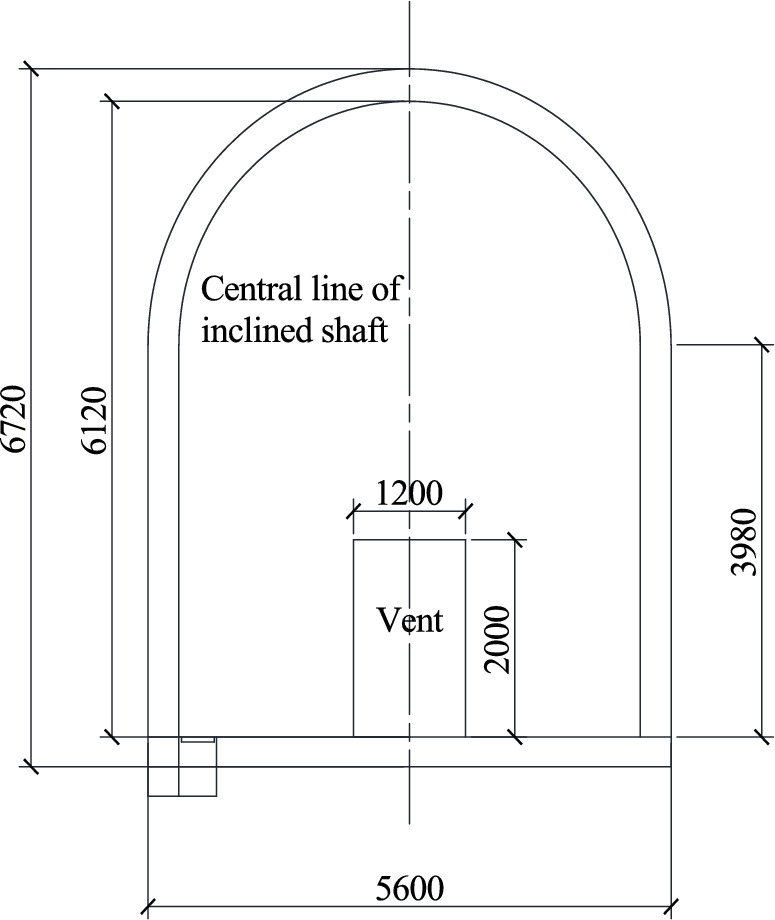
Section of No.5 inclined shaft and position of air intake

**Figure. 20:**
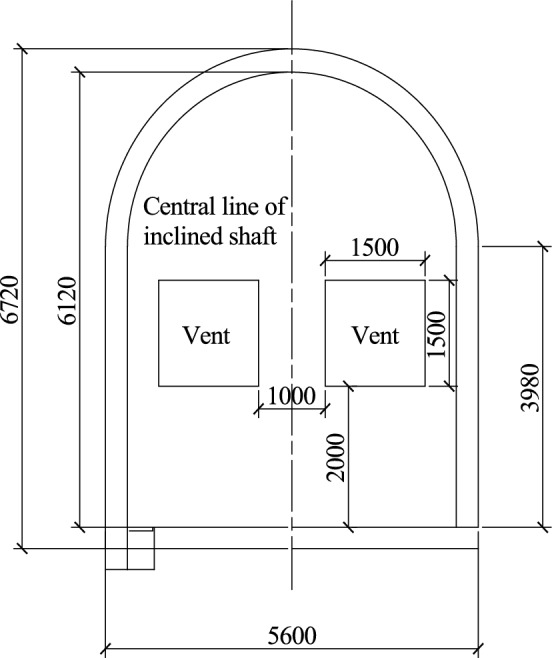
Location of the tuyere at the sealing wall of No. 2 branch hole in inclined shaft No. 5

In the ventilation scheme, inclined shaft No. 5 is closed on one side of the existing tunnel, and only the air intake with a width of 1.2 m and a height of 2 m is retained, as shown in Fig. 19. A sealing wall is set on one side of inclined shaft No. 5 near branch hole No. 2, and only two air intake outlets with a height of 2 m from the ground and a length and width of 1.5 m are reserved. Two axial flow fans are arranged outside the air intake outlets, as shown in Fig. 20. In order to explore the influence of air volume and inclination Angle of inclined shaft on overturning coefficient of train, air volume in actual condition and virtual condition were set for each inclined shaft. The air volume of the inclined shaft is shown in Table 9.

**Table 9:** Air intake from inclined shaft

Inclined shaft	No. 5 inclined shaft	No. 7 inclined shaft	No. 8 inclined shaft	No. 9 inclined shaft
Actual condition take air volume (m^3^/min)	3024	2946	3157	3095
Virtual condition 1 take air volume (m^3^/min)	1800	1800	1800	1800
Virtual condition 2 take air volume (m^3^/min)	5400	5400	5400	5400
Virtual condition 3 take air volume (m^3^/min)	9000	9000	9000	9000

### Model building

#### Calculation model

**Figure. 21:**
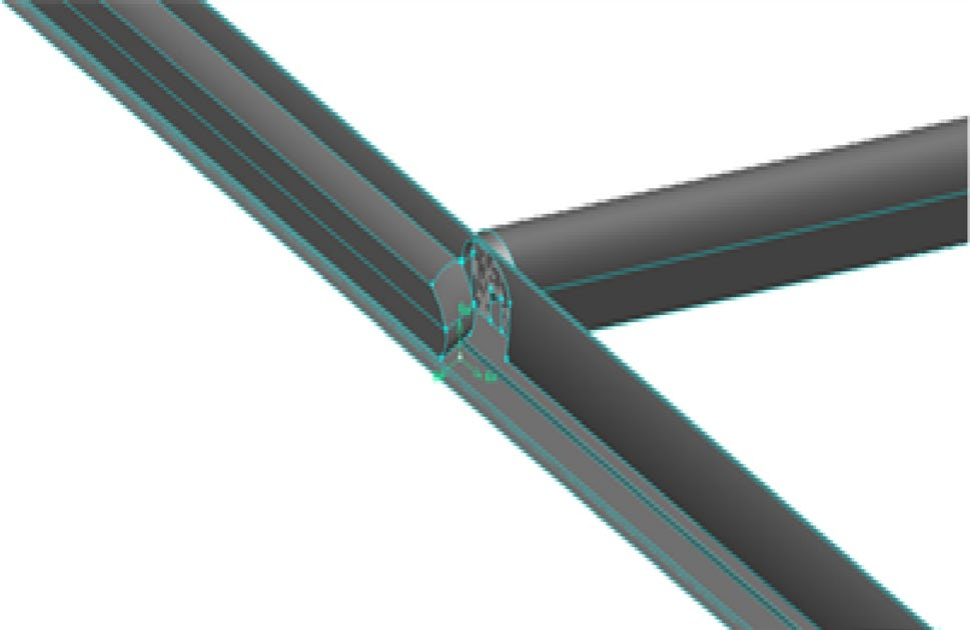
Numerical calculation model

**Figure. 22:**
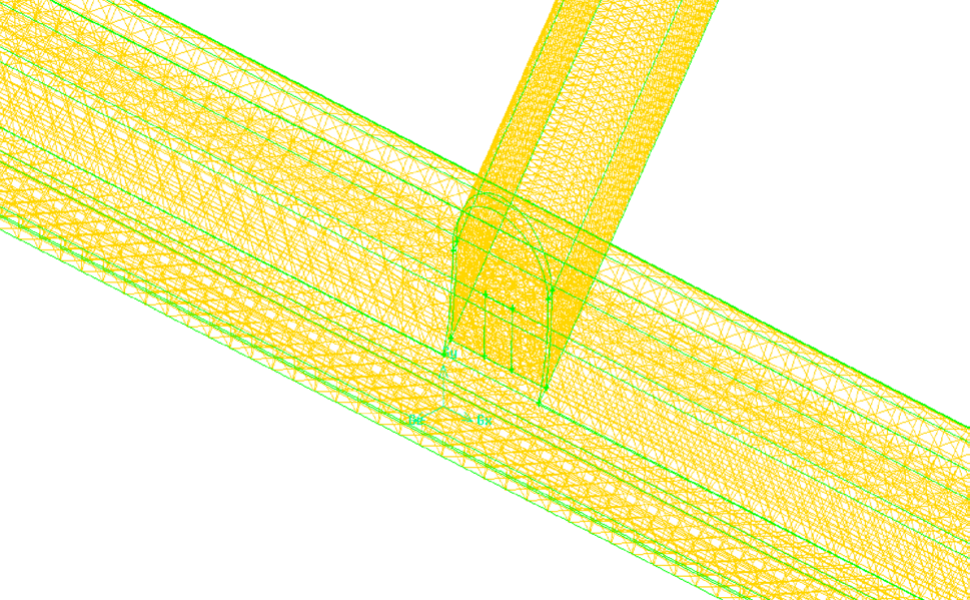
Grid at the intersection of inclined shaft and existing tunnel

Based on the above parameters, a three-dimensional numerical model of a tunnel-train-inclined shaft was established, as shown in Figs. 21 and 22.

#### Point arrangement

Air pressure monitoring points are arranged on the body surface at the middle position of the top of the head car, the middle car and the tail car. These cars are defined in Section "Finite element model" of this paper.

**Figure. 23:**
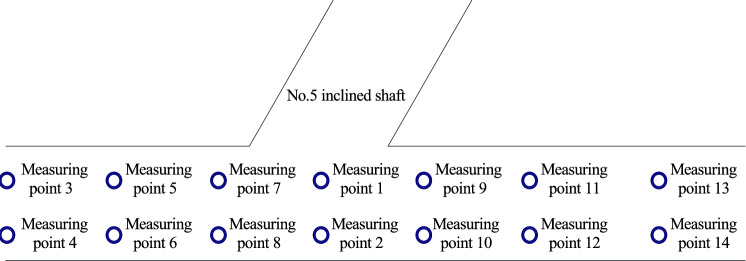
Schematic diagram of location of wind speed measurement points

Wind speed monitoring points are arranged around each inclined shaft, and the measuring points of each inclined shaft are consistent with their relative positions. The measuring point arrangement is described by taking inclined shaft No. 5 as an example. Based on the central axis of the existing tunnel and the centerline of inclined shaft No. 5, 14 wind speed measurement points were symmetrically arranged and numbered 1–14. Among these points, measuring points 1, 3, 5, 7, 9, 11 and 13 are located 2.05 m from the central axis of the existing tunnel on the left side of the inbound direction (0.5 m from the vehicle body), and measuring points 2, 4, 6, 8, 10, 12 and 14 are located on the right side of the inbound direction and are symmetrically arranged along the central axis of the existing tunnel. The longitudinal distances from the centerline of the inclined shaft are 0 m, ± 2.5 m, ± 5 m and ± 10 m, and the height of the measuring points is 2 m. The locations of the wind speed measurement points are shown in Fig. 23.

### Calculation results and safety evaluation

#### Train operating comfort

The sealing index of HXD1D trains operating in the existing Wushaoling Tunnel is approximately 3 s, and the maximum transient pressure inside the front, middle and rear vehicles is calculated, as shown in Table 10.

**Table 10:** Maximum 3s transient pressure inside the train

Item	Head car	Middle car	Tail car
Maximum in-car value (Pa/3 s)	463.07	470.53	469.40

As shown in Table 10, the maximum 3 s transient pressure in the three carriages is lower than that in the previous working conditions, mainly because the train operating speed in the existing Wushaoling Tunnel is relatively low—only 120 km. In the existing Wushaoling Tunnel, the maximum 3 s transient pressure inside the head and middle and rear trains does not exceed 800 Pa, which meets the comfort requirements.

#### Train operation safety

This section mainly evaluates the operational safety of trains in the existing Wushaoling Tunnel from the wind speed and the overturning coefficient.

(1) Wind speed

(1) Demand

According to the relevant requirements of the “Railway Technical Management Regulations (High-speed Railway Part)”, the speed requirements of trains are given, as shown in Table 11 below.

**Table 11:** Wind speed

Wind speed	Status
*v* ≤ 15 m/s	Normal operation
15 m/s<*v* ≤ 20 m/s	The operating speed is not more than 300 km/h
20 m/s<*v* ≤25 m/s	The operating speed is not more than 200 km/h
25 m/s<*v* ≤ 30 m/s	The operating speed is not more than 120 km/h
*v* > 30 m/s	Emu trains are strictly prohibited

(2) Calculation results

The wind speeds at different inclined shaft locations are obtained according to the numerical simulation results shown in Table 12.

**Table 12:** Maximum wind speed at different inclined shaft locations (unit: m/s)

Inclined shaft	The fan is on and no train passes	The fan is off and the train is passing	The fan is on and the train is passing
5#	11.2	17.6	26.1
7#	10.3	14.7	18.2
8#	11.8	16.8	19.9
9#	10.7	13.4	22.1

As shown in Table 12, when no train is in the tunnel, air extraction from the inclined shaft slightly influences the flow field in the tunnel, and the maximum wind speed ranges from 10.0 to 12.0 m/s. When the train passes through the tunnel and the inclined shaft does not take air, a large-scale vortex is formed at the inclined shaft air outlet. Because the amount of air squeezed into the inclined shaft when the train passes through is not large, the internal vortex is small, and the maximum wind speed is 13.0–18.0 m/s. When the train passes through the tunnel and receives air from the inclined shaft, the vorticity around the train decreases, and the vorticity amplitude is alleviated to a certain extent. The vorticity on the side near the inclined shaft tends to develop toward the air outlet, and a high-intensity vorticity is formed at the air outlet inside the inclined shaft, with the maximum wind speed ranging from 18.0 to 16.1 m/s. According to the train speed and wind speed requirements, trains can run normally.

(2) Overturning coefficient

Table 13 shows the maximum overturning coefficients of the operating trains at different inclined shaft locations, calculated by substituting the numerical calculation results into Formulas (1)–(7).

**Table 13:** Calculation results of overturning coefficient

Inclined shaft	Angle with the right line (°)	Actual condition overturning coefficient	Virtual condition 1 overturning coefficient	Virtual condition 2 overturning coefficient	Virtual condition 3 overturning coefficient
No. 5	45	0.28	0.18	0.47	0.80
No. 7	59	0.35	0.19	0.64	1.01
No. 8	59	0.37	0.20	0.63	1.07
No. 9	79	0.49	0.30	0.95	1.71

Table 13 shows that the maximum overturning coefficients are less than 0.8, meeting the specification requirements. Therefore, train operation is safe. The larger the relative angle between the inclined shaft and the existing tunnel and the larger the air volume, the higher the overturning coefficient of the train. The overturning coefficient is approximately inversely proportional to the angle and approximately proportional to the air volume. It can be seen that the air volume should be limited in the inclined shaft to ensure the safety of the train.

## Conclusion

Considering that taking air from the inclined shaft of an existing tunnel affects the comfort and safety of train operation, the change laws of train transient pressure and aerodynamic force (lift coefficient, transverse force coefficient and overturning moment coefficient) were studied, and the change laws of train transient pressure and aerodynamic force under three operating conditions—the inclined shaft closed, the inclined shaft open but not taking air, and the inclined shaft open and taking air—were analyzed. The influence of air extraction from inclined shafts on the comfort and safety of train operation was studied. The following conclusions can be drawn from this research.

1. When the train passes through the tunnel, the negative pressure on the surface of the train body is greater than the positive pressure. Opening the inclined shaft reduces the maximum negative pressure of the operating train. When the inclined shaft is open, the maximum negative pressures at the measuring points on the head car, middle car and tail car are reduced by 23.0%, 20.3% and 15.4%, respectively, when no air is used and by 23.0%, 20.3% and 15.4%, respectively, when air is used
2. The presence of an inclined well can reduce the pressure variation amplitude at 3 s in the carriage, and the change in transient pressure in the carriage is not obvious whether or not the air is taken from the inclined well; moreover, the air taken from the inclined well has almost no effect on the comfort of the train.
3. Neither the maximum nor the minimum head and tail car lift coefficients differ considerably under the three working conditions. When the head car reaches the inclined shaft after the inclined shaft is opened, the force on the side of the pointing track suddenly increases and then decreases, exhibiting a “nodding” trend. When the tail car drives from the inclined shaft, the lift force first increases and subsequently decreases within a short time, and a “head-up” trend occurs.
4. The transverse force coefficients under the three working conditions differ greatly when the head car arrives at the inclined shaft, the middle car passes through the inclined shaft and the tail car leaves the inclined shaft. The train suffers an increase in the transverse force while passing through the inclined shaft, increasing its swing tendency. Compared to the condition without taking air, taking air from the inclined shaft increases the swing degree of the head car to the side of the inclined shaft.
5. The overturning moment coefficients of the three working conditions differ greatly when the head car arrives at the inclined shaft and when the tail car leaves the inclined shaft. The possibility of the train rolling increases when it passes through the inclined shaft. Compared to the condition without taking air from the inclined shaft, taking air from the inclined shaft increases the inclination degree of the head car to the side of the inclined shaft.
6. According to the actual engineering calculation results, the maximum wind speed of the four shafts under the most unfavorable working conditions is 26.1 m/s, and the maximum overturning coefficient is 0.49. The air extraction from the inclined shaft meets the aerodynamic safety and comfort requirements of the operating trains in the existing Wushaoling Tunnel.
7. When the operating train enters the tunnel, the air intake of No. 5 inclined shaft should not be greater than 9000 m^3^/min; the intake air volume of Nos. 7 and 8 inclined shafts should not be greater than 7000 m^3^/min ; the air volume of No. 9 inclined shaft should not be greater than 4500 m^3^/min. If the air volume that meets the construction requirements is greater than the limit, the train is prohibited from entering the tunnel to avoid overturning, and the tunnel can only be entered when the construction is completed or the air volume is less than the limit.

## Data Availability

All data generated or analysed during this study are included in this published article [and its supplementary information files].

## References

[1] Xiang CQ, Guo WH, Zhang JW (2014). Overturning stability of a high-speed train running on a bridge and optimal height of wind barriers under strong crosswind. J. Vib. Shock.

[2] Du JJ, Wu YC, Guan HN (2021). Influence of fluctuating crosswind on the ride quality of high-speed train. J. Chongqing Univ. Technol. (Nat. Sci.).

[3] Luo JB, Hu YY, Yang JH (2013). Effect of embankment inclining angle on aerodynamic characteristics of high speed train under crosswinds. Chin. J. Comput. Phys..

[4] Xie FR, Jin AF, Li H (2022). Influence of sand: Carrying cross wind under different embankments on aerodynamic characteristics of high speed trains. Mach. Tool Hydraul..

[5] Liu R, Yao S, Xv JE (2019). Study on critical overturning wind speed of high-speed train under cross wind. J. Railw. Sci. Eng..

[6] Ezoji R, Talaee MR (2021). Analysis of overturn of high-speed train with various nose shapes under crosswind. Iran. J. Sci. Technol. Trans. Mech. Eng..

[7] Cheli F, Ripamonti F, Rocchi D (2010). Aerodynamic behaviour investigation of the new EMUV250 train to cross wind. J. Wind Eng. Ind. Aerodyn..

[8] Zhou D, Yu D, Wu L (2023). Numerical investigation of the evolution of aerodynamic behaviour when a high-speed train accelerates under crosswind conditions. Alex. Eng. J..

[9] Montenegro PA, Heleno R, Carvalho H (2020). A comparative study on the running safety of trains subjected to crosswinds simulated with different wind models. J. Wind Eng. Ind. Aerodyn..

[10] Niu J, Zhou D, Liu T (2017). Numerical simulation of aerodynamic performance of a couple multiple units high-speed train. Veh. Syst. Dyn..

[11] Yu M, Liu J, Liu D (2016). Investigation of aerodynamic effects on the high-speed train exposed to longitudinal and lateral wind velocities. J. Fluids Struct..

[12] Zhuang Y, Lu X (2015). Numerical investigation on the aerodynamics of a simplified high-speed train under crosswinds. Theor. Appl. Mech. Lett..

[13] Proppe C, Zhang X (2015). Influence of uncertainties on crosswind stability of vehicles. Proc. Iutam.

[14] Taiming H, Ma JM, Zhang L (2023). The influence of vehicle body roll motion on aerodynamic characteristics under crosswind condition. Int. J. Numer. Methods Heat Fluid Flow.

[15] Morden JA, Hemida H, Baker CJ (2015). Comparison of RANS and detached eddy simulation results to wind-tunnel data for the surface pressures upon a class 43 high-speed train. J. Fluids Eng..

[16] Li T, Zhang J, Zhang W (2013). A numerical approach to the interaction between airflow and a high-speed train subjected to crosswind. J. Zhejiang Univ. Sci. A.

[17] Zhang L, Yang M, Liang X (2017). Oblique tunnel portal effects on train and tunnel aerodynamics based on moving model tests. J. Wind Eng. Ind. Aerodyn..

[18] Niu J, Liang X, Zhou D (2016). Experimental study on the effect of Reynolds number on aerodynamic performance of high-speed train with and without yaw angle. J. Wind Eng. Ind. Aerodyn..

[19] Gao H, Liu T, Gu H (2021). Full-scale tests of unsteady aerodynamic loads and pressure distribution on fast trains in crosswinds. Measurement.

[20] Liu D, Wang T, Liang X (2020). High-speed train overturning safety under varying wind speed conditions. J. Wind Eng. Ind. Aerodyn..

[21] Bell JR, Burton D, Thompson M (2014). Wind tunnel analysis of the slipstream and wake of a high-speed train. J. Wind Eng. Ind. Aerodyn..

[22] Gilbert, T., Baker, C., Quinn, A., & Sterling, M. Aerodynamics of high-speed trains in confined spaces. In *Proceedings of the 7th International Colloquium on Bluff Body Aerodynamics and Applications* 10 (2012).

[23] Tian HQ (2019). Review of research on high-speed railway aerodynamics in China. Transp. Saf. Environ..

[24] Allain E, Paradot N (2014). Aerodynamics in train cross wind studies. Int. J. Aerodyn..

